# Keratin 7 Is a Constituent of the Keratin Network in Mouse Pancreatic Islets and Is Upregulated in Experimental Diabetes

**DOI:** 10.3390/ijms22157784

**Published:** 2021-07-21

**Authors:** Catharina M. Alam, Sarah Baghestani, Ada Pajari, M. Bishr Omary, Diana M. Toivola

**Affiliations:** 1 Department of Biosciences, Cell Biology, Åbo Akademi University, Tykistökatu 6A, BioCity 2nd Floor, FIN-20520 Turku, Finland; sarah.baghestani@abo.fi (S.B.); ada.pajari@abo.fi (A.P.); 2 Center for Advanced Biotechnology and Medicine, Rutgers University, Piscataway, NJ 08854, USA; bo163@cabm.rutgers.edu; 3 Turku Center for Disease Modeling, University of Turku, Kiinamyllynkatu 10, FIN-20520 Turku, Finland

**Keywords:** beta-cell, islet, keratin, *Krt7*, diabetes, stress, polarity, MIN6

## Abstract

Keratin (K) 7 is an intermediate filament protein expressed in ducts and glands of simple epithelial organs and in urothelial tissues. In the pancreas, K7 is expressed in exocrine ducts, and apico-laterally in acinar cells. Here, we report K7 expression with K8 and K18 in the endocrine islets of Langerhans in mice. K7 filament formation in islet and MIN6 β-cells is dependent on the presence and levels of K18. K18-knockout (K18^‒/‒^) mice have undetectable islet K7 and K8 proteins, while K7 and K18 are downregulated in K8^‒/‒^ islets. K7, akin to F-actin, is concentrated at the apical vertex of β-cells in wild-type mice and along the lateral membrane, in addition to forming a fine cytoplasmic network. In K8^‒/‒^ β-cells, apical K7 remains, but lateral keratin bundles are displaced and cytoplasmic filaments are scarce. Islet K7, rather than K8, is increased in K18 over-expressing mice and the K18-R90C mutation disrupts K7 filaments in mouse β-cells and in MIN6 cells. Notably, islet K7 filament networks significantly increase and expand in the perinuclear regions when examined in the streptozotocin diabetes model. Hence, K7 represents a significant component of the murine islet keratin network and becomes markedly upregulated during experimental diabetes.

## 1. Introduction

Keratin (K) 7 is a type II intermediate filament (IF) protein expressed primarily in simple-type epithelia of glandular and ductal cells, but it is also expressed in stratified and transitional epithelia of urothelial and mesothelial tissues [1,2]. In mouse tissues, K7 is widely expressed in the ductal cells of the liver, kidney, and pancreas, and in subpopulations of cells in gastrointestinal and lung epithelia [3]. K7 has a close protein sequence similarity with K8 (77%; www.ebi.ac.uk/Tools/psa/ (accessed on 1 May 2021)), and these two type II keratins are often expressed in the same epithelial tissues with type I keratins K18 and/or K19 and sometimes K20. While K8 is expressed broadly in most simple-type epithelial cells and is well-studied in digestive organs, K7 expression is generally restricted to cell subpopulations within these tissues, such as the base of colonic crypts and cholangiocytes. In the pancreas, K8 and K18 have been described as the major keratins in exocrine acinar cells, as well as endocrine islets [4,5,6,7]. K7 and K19 expression is limited to the ducts and the acinar apico-lateral domain in mouse pancreas, and in some human and rodent studies, K7 expression has been described exclusively in the exocrine pancreatic ducts, where it pairs with the type I K19 [3,5]. However, some species differences have been described in the expression of pancreatic K19, and K19 and K20 expression can be induced in response to exocrine pancreas injury [8,9,10]. K7 immunostaining has been observed in adult pancreatic rat islets, albeit to a lower degree than in ducts [11], and in islet cells at early stages of porcine development [12]. Hence, K7 is generally considered a ductal marker in the pancreas and liver in humans and experimental mammalian models, with some reports describing its presence in pancreatic islets.

Studies using K8-knockout (K8^‒/‒^) mice have shown that keratins play a role in β-cell function. The loss of K8 leads to lower pancreatic insulin content, disrupted insulin vesicle morphology, and abnormal blood glucose regulation [13]. In addition, β-cell mitochondria of K8^‒/‒^ mice are smaller, display increased motility, as well as decreased membrane potential and ATP production [14]. In wild-type streptozotocin (STZ)-treated and non-obese diabetic (NOD) mice, the β-cell K8 network density increased, suggesting that keratins may have a function in protecting β-cells from diabetes-induced cell stress or injury [13]. In concordance with this, mice expressing only one K8 allele were more sensitive to STZ-induced diabetes. Meanwhile K8^‒/‒^ mice are protected from acute STZ-induced β-cell injury, likely due to mistargeting of the glucose transporter 2 (GLUT2) from the plasma membrane to the cytoplasm. However, K8^‒/‒^ mice manifest significant endocrine and exocrine pancreatic injury after chronic low-dose STZ treatment [13]. Notably, K8^‒/‒^ mice retain low levels of islet K18 expression, which co-localizes with sparsely expressed K7 filaments [13]. Moreover, K7 protein expression in isolated mouse islets has been detected in a study of the islet proteome [15]. However, the level and location of K7 expression, and its contribution to islet and β-cell IF networks are not known. In this study, we: (i) examine the keratin composition of mouse β-cells, with a focus on K7 expression, keratin filament partnering, and subcellular distribution within the polarized β-cell rosette [16,17], and (ii) assess keratin expression patterns during diabetic stress.

## 2. Results

### 2.1. Type II K7 Is Expressed in Murine Islets In Vivo Together with Type II K8 and Type I K18, and Its Expression Is Dependent on the Presence and Levels of K18

**Figure 1:**
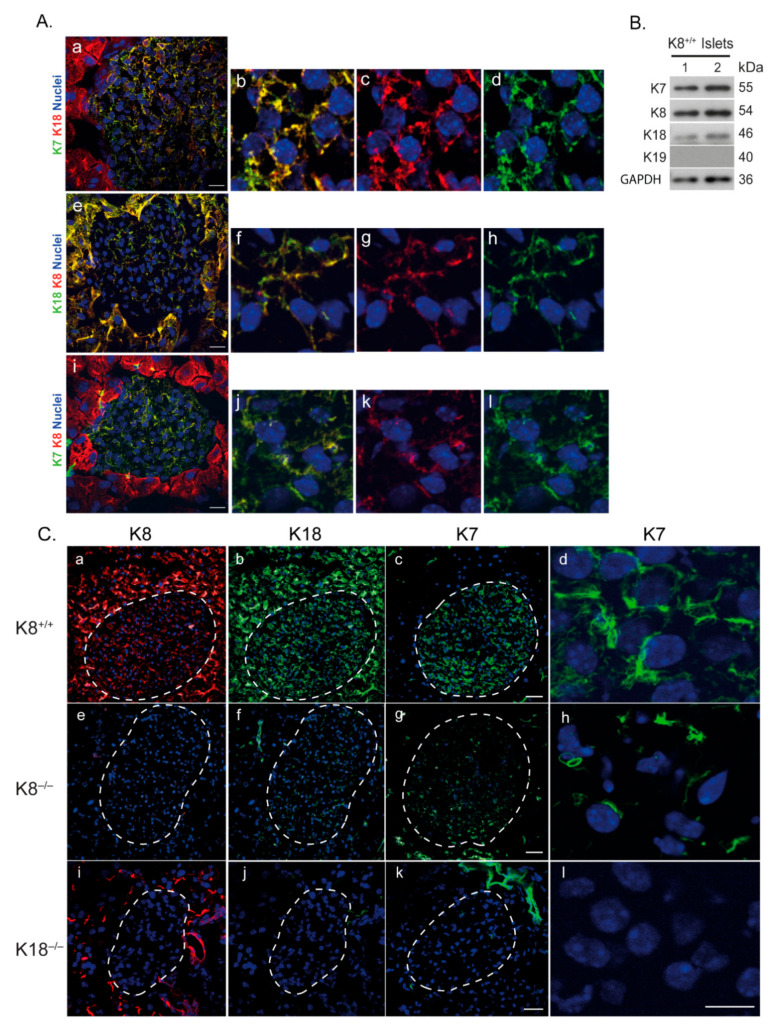
K7 is expressed in pancreatic islets alongside K8 and K18 and its expression is dependent on K18 levels. (**A**) Wild-type K8^+/+^ mouse pancreatic tissue sections were immunostained for K7, K8, K18 and nuclei. The merged images of double staining (**a**,**e**,**i**) as well as the individual separate images labelling K7, K8 and K18 (**b**–**d**,**f**–**h**,**j**–**l**, show co-expression of K7/K8 and K18 filaments in islets. Scale bar = 20 μm. (**B**) Protein lysate from pancreatic islet cells isolated from wild-type K8^+/+^ mice (lanes 1–2) were immunoblotted for K7, K8, K18, K19 and GAPDH (loading control). Each lane contains islets isolated from 2 mice. (**C**) Immunostaining of pancreatic sections from K8^+/+^ (**a**–**d**), K8^−/−^ (**e**–**h**) and K18^−/−^ (**i**–**l**) mice for K8, K18 and K7, showed a reduction in K7 expression in K8^−/−^ islets (**h**) and total absence of K7 in mice lacking K18 (**l**). Scale bar = 50 μm (in **c**,**g**,**k** for **a**–**c**,**e**–**g**,**i**–**k**) and 20 μm (for **d**,**h**,**l**). *n* = 3 mice per genotype.

Since K8^‒/‒^ mouse endocrine islets express residual type I K18 filaments [13], we examined the presence and level of islet type II K7. Immunostaining of wild-type mouse pancreas sections (Figure 1A) and immunoblot analysis of isolated islets (Figure 1B) showed that, in addition to K8 and K18, pancreatic islets in mice express substantial amounts of K7 which co-localizes with K8 and K18 (Figure 1A). In exocrine pancreatic ducts, K7 is expressed with K19 (Figure 1C(k), Supplementary Materials Figure S1). However, K19 was not detected in wild-type K8^+/+^ or K8^‒/‒^ islets (Figure 1B, Supplementary Materials Figure S1A). 

The K7 expression within the islets is dependent on the presence and levels of K18. K8^‒/‒^ animals have significantly reduced pancreatic K18 levels and show concomitant K7 downregulation and disruption of K7 filament organization (Figure 1C(e–h)). Importantly, K18-knockout (K18^‒/‒^) mice completely lack K7 and K8 filaments in the islets (Figure 1C(i–l)). Exocrine ducts are K7/K19 positive in these sections (Figure 1C(k)) and K8 is present in the apical part of acinar cells (Figure 1C(i)) due to pairing with K19 [8,18], verifying that the stainings were successful. Furthermore, immunostaining of wild-type pancreatic islets showed that K7 is also present in glucagon producing α-cells (Supplementary Materials Figure S2). Taken together, these results show that K18 is the only type I keratin in β-cells and that it forms filaments with K8 and K7. 

### 2.2. Keratin Expression in Islets Is Enriched Near the β-Cell Vertices and Lateral Edges

**Figure 2:**
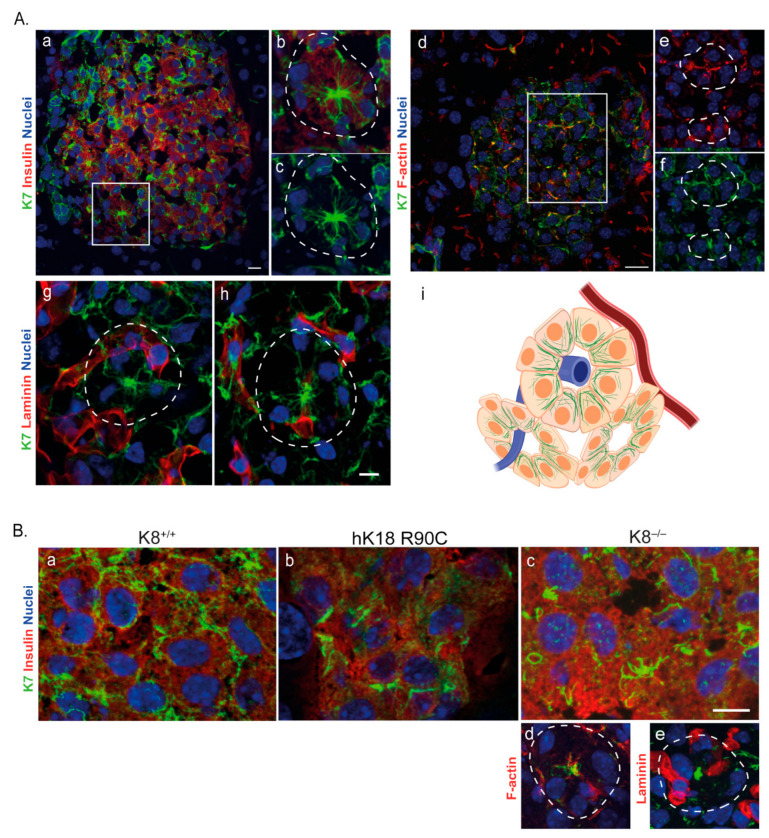
K7 is mainly expressed apicolaterally in K8^+/+^ islet β-cells, as well showing a cytoplasmic filament network which is fragmented in the absence of K8 (K8^−/−^) or disruption of K18 (hK18 R90C) filaments. (**A**) Pancreatic tissue sections from K8^+/+^ mice were immunostained for K7 (green), insulin (red) and nuclei (blue). The merged image of double staining of islet (**a**) and the higher magnification individual separate images of the islet rosette (**b**,**c**), showed the K7 expression as thick bundles near the apical (center of rosette) and lateral side of β-cells. The immunostaining of islets for K7 (green) and F-actin (red) in merged image (**d**) and individual higher magnification rosettes (**e**,**f**), showed a similar enrichment in apicolateral side of the cells for K7 and F-actin. The immunostaining of islets for K7 (green) and laminin (red) in higher magnification rosettes (**g**,**h**), showed that K7 filaments are not found on the basal side of β-cells (identified by the laminin-staining). Boxes in a and d indicate areas enlarged in b, c, and e, f respectively. Dotted lines indicate rosettes. Scale bar = 20 μm. The schematic (**i**) shows the K7 β-cell localization in islet rosettes. The venule (blue) in the center and arteriole (red) near the periphery of the rosettes are indicated. K7 filament bundles (green) are enriched in the apical side towards the center of the rosette and the lateral side of β-cells, while are away from the basal side on the periphery of the rosette. (**B**) Pancreatic β-cells stained for K7 (green), insulin (red) and nuclei (blue) in hK18 R90C mouse islets (**b**), showed that K7 was mostly seen as dot-like structures with only scarce filaments. Lateral and cytoplasmic K7 filaments in K8^−/−^ mice (**c**–**e**), were also fragmented compared to K8^+/+^ (**a**). Dotted lines indicate rosettes. Scale bar = 10 μm. *n* = 2 mice per genotype.

In polarized epithelial cells, such as colonocytes and exocrine pancreatic acinar cells, keratin filaments are concentrated at the apical domain, with thinner filament formation along the lateral domains and in the cytoplasm [18,19]. Hepatocytes on the other hand, form several small apical domains at their bile canaliculi [20,21] and the hepatocyte keratin network extends more homogenously from the nucleus outwards, across the cytoplasm [12]. Given the discrete polarized nature of β-cells, we investigated whether the K7 network in β-cells displays a polarized distribution, or whether keratin filaments are homogenously expressed throughout the cytoplasm. We also studied how the filament network dynamics is affected by the absence or mutation of K8 or K18. In immunostainings of mouse pancreas sections, the K7 filaments in wild-type β-cells formed thick bundles near F-actin at the apical/vertex side of the cell, which constitutes the center of the β-cell ‘rosette’ structures [17,22,23], as well as along the lateral domains of the β-cells (Figure 2A(a–f,i)). In contrast, K7 staining was negative near the basal side of the cells identified by laminin staining (Figure 2A(g–h)). In the β-cell cytoplasm, a connected but weaker network of K7, K8, and K18 was seen (Figure 2A). 

In mice expressing the filament assembly-deficient hK18 R90C mutation, K7 was mostly detected as dot-like structures, as has been reported for this mutation in other organs [24,25,26,27], with only scarce cytoplasmic filaments (Figure 2B(b)). These findings indicate that intact K18 is required for maintaining the keratin network morphology. In K8^‒/‒^ islets, most lateral K7/K18 bundles were lost, and the cytoplasmic network was fragmented, with remnant K7 at the apical domain and forming occasional circular structures within the cells (Figure 2B(c–e)). These results suggest, that though some K7 expression remains in K8^‒/‒^ β-cells, it is not sufficient for maintaining the lateral domain filament localization or the preservation of a continuous cytosolic keratin network. This further underlines the indispensability of K18 in β-cells.

### 2.3. Over-Expression of Human K18 in Mice Induces K7 Upregulation

**Figure 3:**
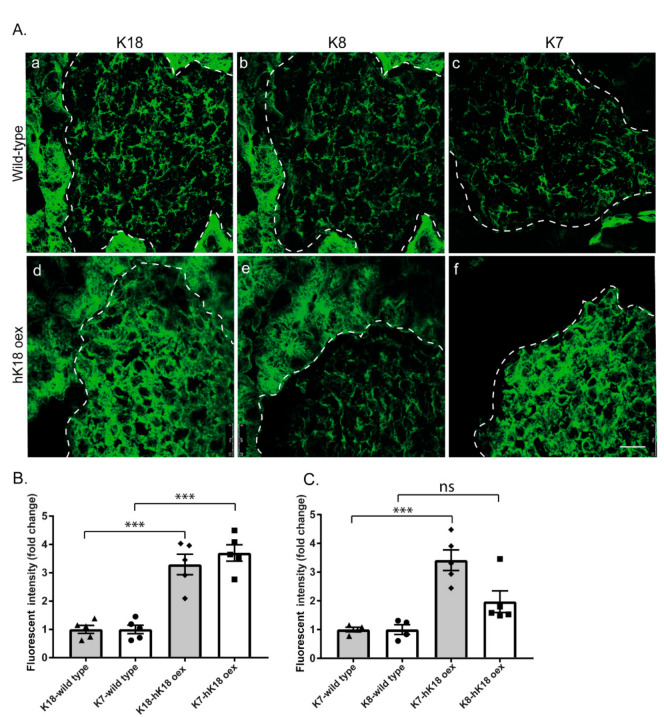
Over-expression of hK18 leads to significant K7 upregulation compared to K8 in mouse pancreatic islets. (**A**) Pancreatic tissue sections of wild-type and hK18 over-expressing mice (hK18 oex) were immunostained for K7, K8 and K18. The individual images labelling keratin filaments in wild-type (**a**–**c**) and hK18 oex (**d**–**f**), showed the increase of K18 expression (**d**) as well as a disproportional increase in K7 expression (**f**), compared to K8 (**e**) in islets of hK18 oex as opposed to wild-type islets. Islets are outlined by dotted lines. Scale bar = 50 μm. (**B**) Quantification of islet K7 and K18 levels from images of immunostained pancreas tissue, indicated a homogenous increase in K7 expression as K18 in hK18 oex mice while (**C**) quantification of K7 and K8 fluorescent intensity, showed a significant increase in K7 level in hK18 oex compared to wild-type and no significant (ns) increase in islet K8. *n* = 2 mice per genotype with minimally 4 islets analyzed per mouse. *** *p* < 0.001, *t*-test is used and error bars represent means ± SEM.

To further test the correlation between K18 and K7, we analyzed whether K18 overexpression affects islet K7 and K8 levels. For this, we used mice that over-express human K18 (hK18) [28]. Islet keratin levels were quantified (Figure 3B,C) from pancreas tissue immunostainings of wild-type and hK18 over-expressing mice (Figure 3A). K18 expression (including human and mouse K18) was increased three-fold in hK18 over-expressing islets compared to wild-type islets (Figure 3B). K18 expression was accompanied by an identical and statistically significant three-fold increase of K7 (Figure 3B), while K8 levels were not significantly increased (Figure 3C). 

### 2.4. MIN6 β-Cells Contain Low Levels of Soluble Non-Filamentous K7 and Over-Expression of K18 Restores the K7 Filament Networks 

**Figure 4:**
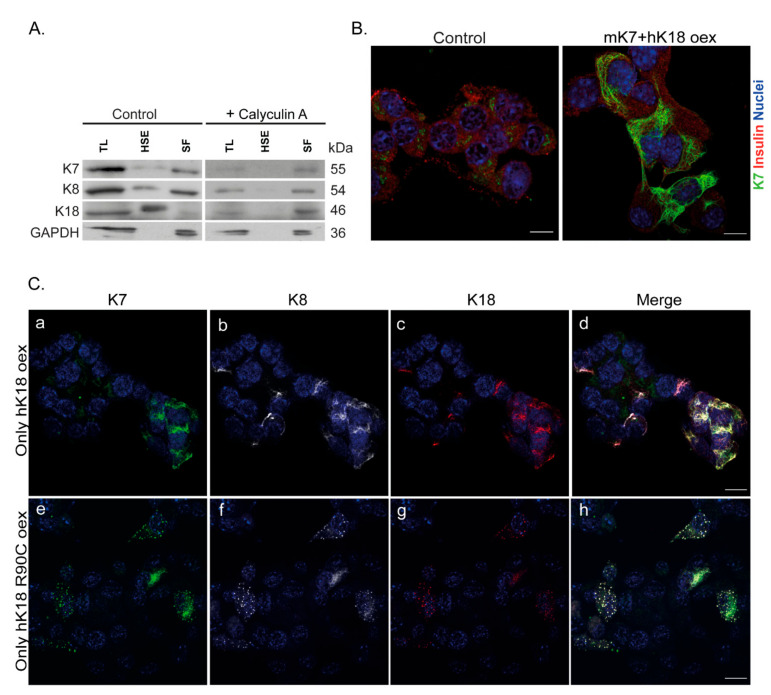
K7 filament formation and levels in MIN6 β-cells is dependent on K18 levels. (**A**) Immunoblots of keratin high salt extraction (HSE) from total lysate (TL) of MIN6 β-cells show the endogenous K7, K8 and K18 in insoluble (HSE) and soluble (SF) form. The soluble K7 fraction could be further increased, if treating the cells with calyculin A. For visualization, the HSE samples were loaded in gel for untreated cells 10-fold, and for calyculin A treated cells 20-fold, more compared to the SF samples. (**B**) MIN6 β-cells transfected with mK7/hK18, formed proper keratin filaments shown in the immunostaining of cells for K7 (green), insulin (red) and nuclei (blue). Scale bar = 10 μm. (**C**) MIN6 β-cells transfected with only hK18 or hK18 R90C (oex) and stained for K7 (green), K8 (grey) and K18 (red). The images (**a**–**d**) show an increased K7/K8/K18 filament formation in cells transfected with hK18. Expressing hK18 R90C in MIN6, lead to similar cytoplasmic K7/K8/K18 dots (**e**–**h**) found in vivo. Scale bar = 20 μm. *n* = 3.

We next investigated the β-cell K7 partner patterns in vitro using MIN6 mouse insulinoma cells. MIN6 cells express very low endogenous levels of K7, K8, and K18 and no K19 (Supplementary Materials Figure S3) and rarely but occasionally form filament-like structures (Supplementary Materials Figure S3B(d–f)). Based on biochemical analysis using high salt extraction (HSE), the scarce endogenous K7, K8, and K18 partition both to the Triton X-100 soluble and non-soluble high-salt fractions (Figure 4A). Phosphatase inhibitor calyculin A treatment, which renders keratins more soluble, was used as a positive control [29] (Figure 4A). Interestingly, K8 and K7 partition more readily to the detergent soluble fraction while K18 partitions mostly to the high salt fraction. The relatively high ratio of K7 and K8 in the soluble fraction could explain the lack of endogenous filament networks in MIN6 cells.

To assess whether MIN6 cells are capable of expressing K7 filaments, we over-expressed mouse K7 (mK7) with hK18 using transient transfection. This resulted in extensive cytoplasmic K7 networks (Figure 4B). Furthermore, over-expressing only hK18, resulted in an increase in endogenous MIN6 K7 and K8 filaments which co-localized with K18 (Figure 4C(a–d)). Similarly, over-expressing the hK18R90C filament assembly-deficient mutation alone, led to cytoplasmic K18 dots that contained endogenous K7/K8, similar to those seen in vivo (Figure 4C(e–h)). The specificity of the anti-K7 antibody used here, a rabbit anti-K7 antibody, was confirmed using a mouse anti-K7 antibody [5,13]. Using immunoblot analysis, both K7 antibodies detected mouse and human K7 at expected molecular weights when over-expressed together with hK18 in MIN6 cells (Supplementary Materials Figure S4A). While the mouse K7 (mK7) antibody did not detect the diffuse non-filamentous K7 by immunostaining, it detected the filament network formed by the overexpression of mK7 or hK7 with hK18 in MIN6 cells similar to the rabbit anti-K7 antibody (Supplementary Materials Figure S4). Taken together, these results show that K7 filament formation is dependent on K18 levels in MIN6 cells similarly as in mouse islets in vivo, and that K7/K8/K18 are co-localized in the same filament bundles.

### 2.5. Islet K7 Becomes Upregulated and Broadly Distributed as Cytoplasmic Filaments in Prediabetic and Diabetic Mice 

**Figure 5:**
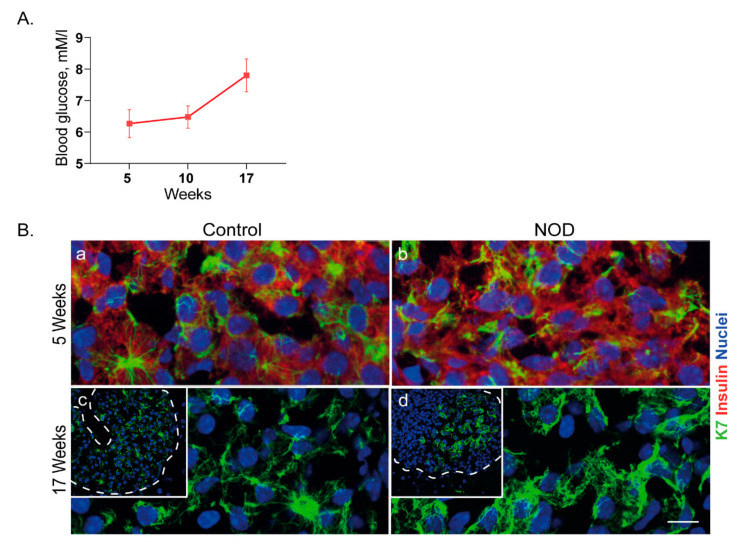
K7 is upregulated in pancreatic islets of prediabetic NOD mice. (**A**) Non-fasting blood glucose levels as a function of time, are shown for NOD mice during 17 weeks follow-up; mean ±SEM. (**B**) Pancreatic tissue sections from control (K8^+/+^) and NOD mice in 5 weeks (**a**,**b**) and 17 weeks (**c**,**d**) of age, were immunostained for K7 (green), insulin (red) and nuclei (blue). The inserts in c and d show lower magnifications of islets (outlined by dotted lines), and the main images in c and d are higher magnification of a section of the shown islets. K7 expression is similar to control in young NOD 5-week old mice, whereas in prediabetic 17-week old mice, K7 is upregulated in some islets. Scale bar = 20 μm. *n* = 2 mice per group.

**Figure 6:**
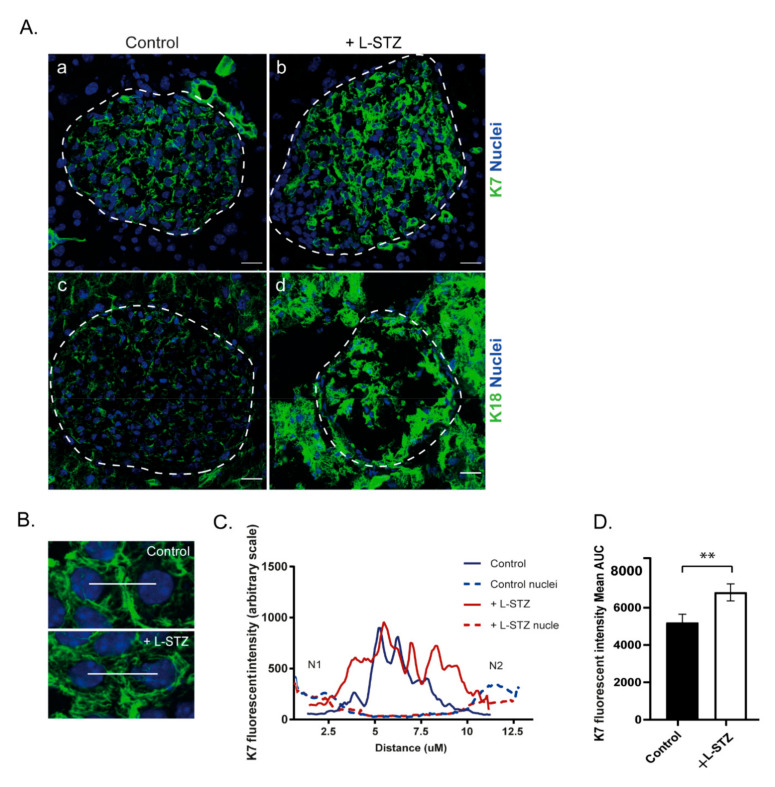
K7 is upregulated and K7 cytoplasmic filaments become more prominent in pancreatic islets upon low-dose streptozotocin treatment. (**A**) Pancreatic tissue sections from control and diabetic low-dose streptozotocin (+L-STZ) treated mice were immunostained for K7, K18 (green) and nuclei (blue). Images show upregulation of K7 (**b**) as well as K18 (**d**) in pancreatic islets upon L-STZ treatment compared to control (**a**,**c**). Islets are outlined by dotted lines. Scale bar = 20 μm. *n* = 2 mice per group with minimally 10 islets analyzed per mouse. (**B**) K7 filament localization in islet β-cells, is analyzed by profiling the K7 intensity along a line connecting the center of nuclei of two neighboring cells in L-STZ treated and control mouse islets. (**C**) Graph representing the K7 intensity, between the nuclei of islet β-cell pairs, *n* = 5 β-cell pairs. (**D**) K7 levels were significantly higher in islets of L-STZ treated mice compared to control demonstrated by the mean area under curve (AUC) for K7, *n* = 50 β-cell pairs. ** *p* < 0.01, using the Student’s t-test. Error bars represent mean ± SEM.

Keratins are upregulated during multiple stress and disease conditions [30], while not much is known about K7 regulation in cell stress. In order to investigate if K7 levels are affected during diabetes stress, we analyzed K7 in prediabetic NOD mice, and mice treated with the diabetes-inducing drug streptozotocin. By immunostaining of pancreas samples from 5-week old newly weaned NOD mice and 17-weekold NOD mice (Figure 5A), we evaluated the islet K7 protein levels in different stages of prediabetic islet stress, compared to control mice. In 5-week old mice, the K7 levels were unaltered compared to controls (Figure 5B(a,b)), while at 17 weeks of age, K7 increased in islets where insulin-expressing cells were still present (Figure 5B(c,d)). K7 levels appeared higher in areas of surviving K7-positive cells in insulin-expressing islets and were observed as more dense and widely distributed cytoplasmic filament networks compared to control mice (Figure 5). Similarly increased K7 and K18 networks and levels were noted 15 days after start of low-dose streptozotocin treatment (Figure 6), coinciding with the onset of full-blown diabetes (blood glucose > 14mMol/L) in these mice [13]. To investigate the cellular K7 level and localization in diabetic conditions, K7 levels and signal distribution were quantified in the cytoplasm of two adjacent β-cells by measuring the fluorescence signal over a line drawn between the two nuclei (Figure 6B). Plotting the K7 fluorescence intensity showed that in diabetic mice, K7 located in a wide area spanning from the peri-nuclear region of one cell, over the plasma membranes, to the peri-nuclear region of the other cell, while K7 in β-cells of control mice, was localized more proximally to the plasma membrane (Figure 6C). Quantifying the total K7 signal in the area between the two cell nuclei (measured as area under the curve) showed a significant increase in total cytoplasmic K7 signal in diabetic β-cells of streptozotocin-treated mice compared to control (Figure 5D). Taken together, K7 is significantly upregulated and expressed more broadly across the entire cytoplasm in diabetic β-cells in vivo, indicating a potential role for K7 in β-cell stress and diabetes.

## 3. Discussion

In contrast to the ubiquitous expression of type II K8 in simple-type epithelia, the type II K7 has a much more limited expression in the body and is often restricted to specific cell localizations or cell types within epithelial tissue. Our results demonstrate that endocrine islets in the mouse pancreas also express substantial levels of K7, in addition to the previously described islet expression of K8 and K18. K7 forms filaments with K18 in the islets, but in the absence of K8, both K7 and K18 are downregulated, likely by proteasomal degradation [31]. Consequently, due to the obligatory heteropolymeric composition of keratins [32], the keratin filament networks become disrupted. This suggests that loss of K8 leads to degradation of K8-bound K18 and some loss of K7 in tetramers that contain 2 K18 + 1 K8 + 1 K7 molecules. In K18-knockout islets, no K7 or K8 can be detected, thereby indicating that K18 is the sole islet type I keratin, and that it pairs with both K7 and K8 to form typical stable filaments. K7 protein levels appear to closely follow K18 protein levels, as K7 rather than K8 is increased in islets from mice over-expressing human K18. However, the over-expression of a type II keratin appears to more robustly drive the expression of the usual type I partner, but less so when a type I is over-expressed [25,28,33,34,35]. This finding aligns well with the results from a proteomics study using C57/Bl6 mice [15] in which K18 was reported to be the most abundant keratin in islets followed by K8 and K7. Notably, the relative abundance score in that study for K18 equaled the sum of the scores for K8 and K7, further implying the combination of keratins in β-cells and the obligate heteropolymeric 1:1 requirement of type II and type I keratins for filament formation and protein stability. A similar K7–K18 correlation was observed in the urothelium of K7-knockout mice, where K7 protein loss was accompanied with a decrease of K18, but not K8, K19, or K20, protein levels [36]. Since K18 was not downregulated on mRNA level, Sandilands et al. suggested that K7 stabilizes K18. In contrast to the K7 expression in mice described here, K7 does not appear to be expressed in human pancreatic islets under basal conditions [37]. The K7 expression in the endocrine pancreas is thus similar to the colon, i.e., species-dependent, as K7 is also expressed in mouse, but not in human colon epithelia, under basal conditions [19,38]. However, while most human colon carcinomas are K7-negative, 5–34% of colon and rectal tumors upregulate and display patchy K7-positive cells, in addition to the reported neoexpression of K7 in various other cancers [19,38]. 

With respect to the keratin filament localization in β-cells, K7/K8/K18 form robust bundles in close proximity to F-actin at the apical and lateral sides, but not towards the basal side of the cell [39]. In β-cells, the cell polarity and functional organization within pancreatic islets is poorly defined, as islet cell clusters lack histologically obvious apical domains [16]. β-cells do, however, form rosette-like structures around islet capillaries and it has been suggested that β-cells make contact with both arterioles and venules [16,17] (see also Figure 2A(i)). These islet ‘rosettes’ lack a clear apical secretory domain, but contain distinct microdomains of functional significance, on the lateral edges and vertices [16,17]. The lateral edges are enriched in functionally essential β-cell signaling and secretory molecules, such as glucose transporter 2 (GLUT2), calcium channels, SNAP-25, and F-actin. Insulin secretion reportedly occurs within discrete apical domains along the lateral side, in areas bordered by tight junctions. Interestingly, GLUT2 becomes mistargeted from the plasma membrane to the cytoplasm in K8^‒/‒^ islets, which have decreased levels of K7/K18 [13], indicating that keratins may be required for protein targeting to the lateral β-cell edges. However, the precise role of islet cytoplasmic or membrane-proximal keratin filaments in GLUT2 targeting remains to be fully investigated. A similarly strong apical distribution of simple epithelial keratins is observed at the subcellular level in exocrine pancreatic acinar cells for K19 and K20, and in intestinal epithelia cells for K8 and K19, and is thought to play a role for essential tissue-specific membrane protein signaling and functions [40]. The accumulation of F-actin and keratins along the lateral edges as well as the β-cell artery-facing vertices suggest a role for the cytoskeleton in maintaining the polarized orientation of β-cells [17].

Therefore, K7, K8, and K18 appear to be the primary type II (K7 and K8) and type I (K18) keratins in β-cells. By contrast with other human tissues where K7 is present, such as in the transitional epithelium of the bladder, K7 is co-expressed with K19, rather than K18 [41]. Similarly, in mice, K7 and K19 are often co-expressed, e.g., in bile ducts, exocrine pancreatic ducts, renal collecting ducts, intestinal epithelial cells, and the bladder epithelium [18,19,36,41,42]. Hence, the mouse islets of Langerhans are, to our knowledge, an exception, as they express K7 but not K19 under basal conditions. Since K7 is expressed uniquely at relatively high levels in mouse islets compared to acinar cells, K7 could be a useful marker, in addition to insulin, for identifying mouse islets in pancreatic sections (Figure 1C, panel c; Figure 3A, panel c; Figure 6A, panel a). Moreover, keratins and other IFs become markedly upregulated in multiple mouse tissues during stress, disease and tissue regenerative conditions [30,43,44,45]). Herein, we show that β-cell K7 levels also increase during murine diabetic stress, similarly to K8 [13]. The increase in K7 levels was observed, especially in the perinuclear region of the β-cells, and appears to occur prior to the development of full-blown diabetes, which raises the hypothesis that K7 may play a role in β-cell pathophysiology or regeneration. 

In conclusion, we report that K7 is a major component of β-cell cytoskeleton in mouse islets of Langerhans, forming filaments with K18 and K8. The K7 containing filaments are concentrated at the apical vertex and along the lateral membranes of β-cells and furthermore form a fine cytoplasmic network. K7 becomes markedly upregulated in the perinuclear regions in experimental mouse diabetes together with K18. In addition, K18 appears to be the only type I keratin in mouse β-cells, and it partners with and stabilizes K7 and K8 to form filaments, while type I K19 is not detected in these cells. The K7–K18 partnership is rather unique as K7 commonly occurs together with K19 in other cell types. The findings in this study highlight the dynamic nature of the simple epithelial keratins, including the less studied K7, in disease and stress conditions, and warrant further studies in human endocrine pancreas pathobiology.

## 4. Materials and Methods

### 4.1. Experimental Animals and In Vivo Experiments 

K8^+/+^ and K8^−/−^ mice of FVB/n background and non-obese diabetic (NOD) mice were bred and raised at the Central Animal Laboratory at the University of Turku. K8^−/−^ mice were bred and genotyped as described in [46]. All animals were used for experiments at 4–7 months of age and were killed by CO_2_ inhalation. Animal experiments performed at the Central Animal Laboratory, University of Turku, were approved by the National Animal Experiment Board (nos. 197/04.10.07/2013 and 3956/04.10.07/2016) and conformed to the regulations set by The Finnish Act on Animal Experimentation. The K18^–/–^ mice, human (h)K18 over-expressing mice, and hK18R90C expressing mice described in [25,28,33,34] were bred in accordance with the Committees on Use and Care of Animals. Streptozotocin (STZ) treatment of K8^+/+^ and K8^−/−^ mice was performed by i.p. administration of 40 mg/kg STZ (Sigma-Aldrich, St. Louis, MO, USA) dissolved in 50 mM sodium citrate buffer (Sigma-Aldrich) in PBS (pH 4.5) in daily doses of 40 mg/kg/d for 5 consecutive days. Blood glucose levels were then monitored daily between day 7 and 14 and animals were considered diabetic when two consecutive measurements exceeded 14 mmol/l [13]. The mice were sacrificed 15 days after the first STZ injection and tissue samples were collected and processed for further analysis. For NOD mice, the pre-diabetic state was confirmed by monitoring of cages for excess urination and blood glucose measurement prior to sacrifice. 

### 4.2. Cell Culture and Transfection 

Murine insulinoma (MIN6) cells (mycoplasma-free; AddexBio, San Diego, CA, USA) were grown in T25 flasks (Sarstedt, Numbrecht, Germany) in Dulbecco’s Modified Eagle Medium (DMEM) supplemented with 15% fetal bovine serum, 1 mM sodium pyruvate, 0.05 mM β-mercaptoethanol, 2 mM L-glutamine, 100 units/mL penicillin, and 100 µg/mL streptomycin under 37 °C and 5% CO_2_. For transfection, the cells were electroporated using the Neon transfection system (Invitrogen, Carlsbad, CA, USA). The cell preparation procedure was performed according to the manufacturer’s instructions and the transfection efficiency (~30%) was optimized following the manufacturer’s recommended protocol. The plasmids including *KRT7* mouse and human untagged clones (OriGene Technologies, Inc. Rockville, MD, USA), *KRT18*; gift from Rudolf Leube, Aachen University, Aachen, Germany and *KRT18 R90C*, gift from Bishr Omary, Rutgers University, New Brunswick, NJ, USA, were inserted using a Neon Gene Pulser (Invitrogen). The transfected cells were then cultured in 24-well plates on glass coverslips in the medium at 37 °C, 5% CO_2_ for 72 h prior to the experiment. For control cells, baseline cells cultured under same conditions were used for cell immunostaining and/or immunoblotting.

### 4.3. Isolation of Islets of Langerhans 

Islets of Langerhans from mice were isolated according to [47], with the exception that 1.5 mg/mL collagenase p (Roche, Mannheim, Germany) was injected through the common bile duct to digest the pancreas. Islets were handpicked under a dissection microscope on ice and cultured in the RPMI-1960 medium supplemented with 10% fetal bovine serum, 2 mM L-glutamine, and 100 U/mL penicillin-streptomycin, for 1–2 days prior to the processing.

### 4.4. High-Salt Extraction 

High-salt extraction (HSE) was performed according to [29]. Cells grown in flasks were 70% confluent at the time of use. In brief, Triton X-100 (Sigma-Aldrich) was added to the freshly harvested cells, mixed, incubated on ice for 2 min and then centrifuged for 10 min at 16,000× *g*. Supernatant was collected and the re-pelleted cells were homogenized manually using a Dounce tissue and cell grinder over ice in high-salt buffer (10 mM Tris-HCl, 140 mM NaCl, 1.5 M KCl, 5 mM EDTA, 0.5% Triton X-100, 1 mM phenylmethylsulfonyl fluoride (Sigma-Aldrich) and complete protease inhibitor cocktail (Roche)). The sample was then incubated/mixed using a rotator for 30 min at 4 °C and centrifuged for 20 min at 16,000× *g*. After washing the pellet using PBS-EDTA, the extract was homogenized for a second time and centrifuged as mentioned above. For the control, half of MIN6 cells in each experiment were treated with 0.2 μM calyculin A for 1 h, harvested, and used for HSE. In parallel, cell total lysates were made by homogenizing freshly harvested cells grown under identical conditions with the cells used for HSE. All the samples were then prepared for immunoblotting by adding non-reducing sample buffer (25 mM Tris-base, 8% Sodium Dodecyl Sulfate, 40% glycerol, 0.02% bromophenol blue, 8.7 mM concentrated phosphoric acid). 

### 4.5. Immunofluorescence Staining and Analysis 

Pancreata from mice were dissected and frozen in tissue embedding O.C.T. compound (Tissue-Tek, Sakura Finetek Europe B.V., Alphen aan den Rijn, The Netherlands), sectioned (6 μm thickness) using Leica CM1950 Research Cryostat (Leica Microsystems, Wetzlar, Germany), and fixed in acetone for 10 min at −20 °C except for F-actin staining for which the sections were fixed in paraformaldehyde (1% vol./vol. in PBS, pH 7.4) for 20 min at RT. Likewise, baseline and transfected MIN6 cells were fixed in acetone for 10 min in −20 °C. The fixed tissue and cell samples were then stained as described in [29], using the following primary antibodies: mouse anti-K7 (monoclonal RCK 105/cat no. 10522, Progen, Heidelberg, Germany), rabbit anti-K7 (monoclonal/ERP17078, Abcam, UK), rat anti-K8 (Troma I; Developmental Studies Hybridoma Bank, Iowa, IA, USA), rabbit anti-K18 (Sigma-Aldrich), mouse anti-K18 (Invitrogen), rat anti-K19 (Troma III; Sigma-Aldrich), rabbit anti-insulin (Cell Signaling, Danvers, MA, USA), mouse anti-insulin (monoclonal; Santa Cruz Biotechnology, Dallas, TX, USA), mouse anti-glucagon (monoclonal; Santa Cruz Biotechnology), rat anti-laminin β 1 (monoclonal; Invitrogen). Secondary antibodies were as follows: donkey anti-rabbit Alexa 488, 546 and 647, donkey anti-rat Alexa 488 and 568, goat anti-mouse Alexa 488 (Invitrogen) and Alexa-Fluor 546 conjugated phalloidin (Invitrogen, Eugene, OR, USA). The nuclei were stained with DRAQ5 (Thermo Fisher Scientific, Bannockburn, IL, USA) or DAPI (Invitrogen) and the stained tissue and cell samples were mounted with ProLong Gold antifade reagent (Invitrogen). The samples were imaged using an SP5 confocal microscope (TCS SP5; Leica) and/or a 3i Marianas Spinning disk confocal microscope (Intelligent Imaging Innovations, Denver, CO, USA). Within each imaging session, all samples were imaged using the same microscopy settings and during image processing, the same adjustments were applied to all images. The quantitative analysis of protein fluorescent intensity and subcellular localization was performed using the open source Fiji distribution of ImageJ software package and/or LAS AF lite confocal software (Leica). For mean fluorescent signal quantification, regions of interest (ROI) were drawn in pancreatic islets and normalized to the size of the ROI, measuring the protein signal intensity per unit area. To obtain the cellular protein signal distribution, a line was drawn between two nuclei of adjacent β-cells to profile the fluorescent intensity along the line. The optical plane was chosen at the plane with the largest nuclear diameter for the cells. 

### 4.6. SDS-PAGE and Immunoblotting

Baseline and transfected MIN6 cells were harvested and homogenized over ice in homogenization buffer (0.187 M Tris-HCl, 3% SDS and 5 mM EDTA, pH 6.8) using a syringe (BD, Franklin Lakes, NJ, USA) and 30 G needle (Henke Sass Wolf, Tuttlingen, Germany). The protein amount in each sample was then determined using Pierce BCA protein assay kit (Thermo Fisher Scientific, Waltham, MA, USA). Thereafter, the samples were prepared for SDS-PAGE and immunoblotting by diluting to 5 µg protein/10 µl using 3× Laemmli sample buffer (30% glycerol, 3% SDS, 0.1875 M Tris-HCl (pH 6.8), 0.015% bromophenol blue and 3% β-mercaptoethanol) unless otherwise stated. The primary antibodies used for immunoblotting were rat anti-K8 (Troma I; Developmental Studies Hybridoma Bank), mouse anti-K7 (monoclonal RCK 105/cat no. 10522, Progen ), rabbit anti-K7 (monoclonal/ERP17078, Abcam, Cambridge, UK), rabbit anti-K18 (Sigma-Aldrich), rat anti-K19 (Troma III; Sigma-Aldrich), rabbit anti-GAPDH (Abcam), and rat anti-Hsc70 (Stressgen Bioreagents, Ann Arbor, MI, USA). The following secondary antibodies were used: anti-rat horseradish peroxidase (HRP) (Cell signaling), anti-rabbit HRP (Promega Biosciences, San Luis Obispo, CA, USA) and anti-mouse HRP (GE Healthcare, Little Chalfont, UK). The blots were then developed using Amersham ECL developing solution (GE Healthcare) and/or Lightning Plus-ECL (Perkin Elmer, Waltham, MA, USA) and exposed to X-ray films (Super RX, Fuji Corporation, Valhalla, NY, USA) for visualization. 

## Data Availability

Not applicable.

## Supplementary Materials

The following are available online at https://www.mdpi.com/article/10.3390/ijms22157784/s1.

## Statistical Analysis

Student’s *t*-test was used when applicable using Prism 6 (GraphPad, San Diego, CA, USA).
