# Supplementary material for: Keratin 7 Is a Constituent of the Keratin Network in Mouse Pancreatic Islets and Is Upregulated in Experimental Diabetes
Source: Int J Mol Sci. 2021 Jul 21;22(15):7784. doi: 10.3390/ijms22157784 (PMC8346022; doi:10.3390/ijms22157784)
Supplement: Supplementary file 1 [file ijms-22-07784-s001.zip › ijms-1262983-supplementary.pdf]

Supplementary figures and legends

Keratin 7 is a constituent of the keratin network in mouse pancreatic islets and is upregulated in experimental diabetes

C.M. Alam*[1], S. Baghestani*[1], A. Pajari[1], M.B. Omary[2], D.M. Toivola[1,3]

[1]Department of Biosciences, Cell biology, Åbo Akademi University, Tykistökatu 6A, FIN_20520 Turku, Finland [2]Center for Advanced Biotechnology and Medicine, Rutgers University, Piscataway, NJ 08854, USA, [3]Turku Center for Disease Modeling, University of Turku, Kiinamyllynkatu 10, FIN-20520 Turku, Finland

[*] equal contribution

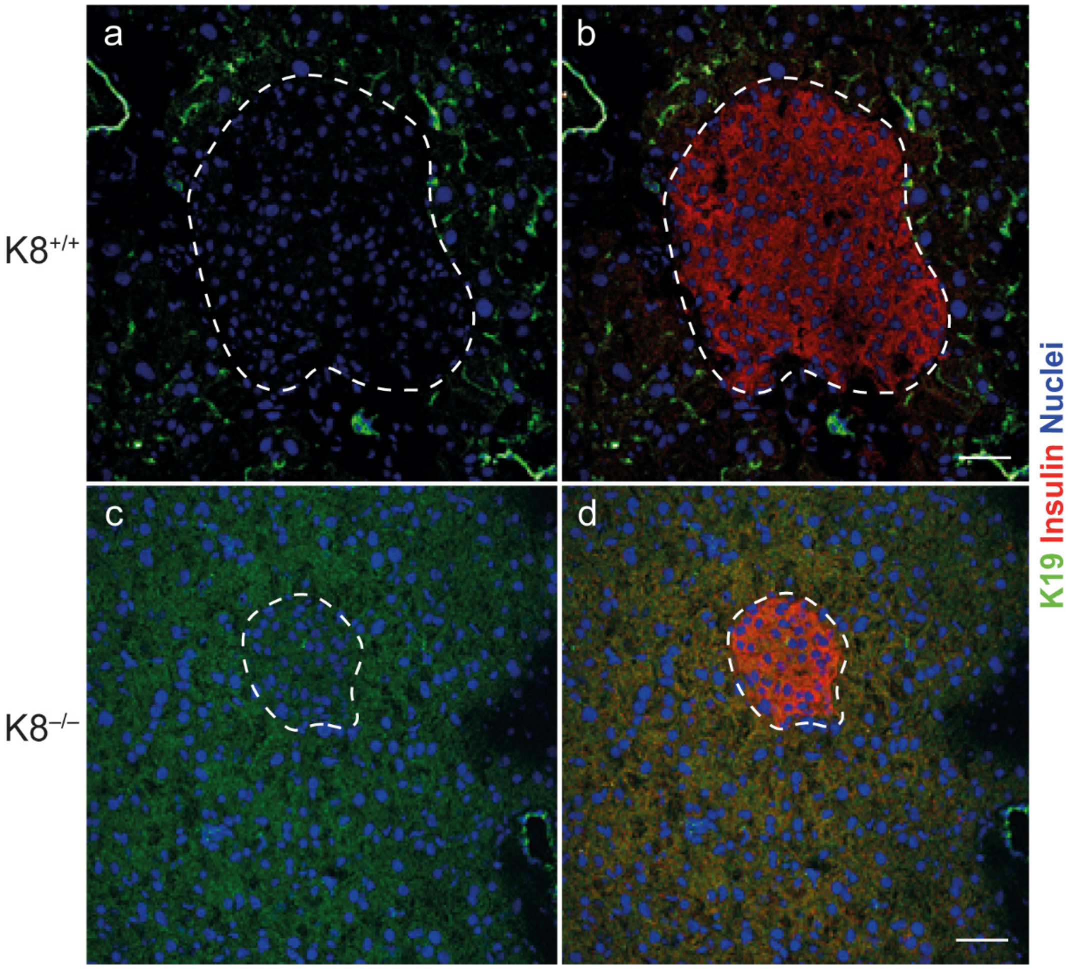

Supplementary Figure S1. K19 is not expressed in pancreatic islets of K8$^{+/+}$ and K8$^{–/–}$ mice. Immunostaining of pancreatic tissue sections from K8$^{+/+}$ (a, b) and K8$^{–/–}$ (c, d) mice for K19 (green), insulin (red) and nuclei (blue), show that K19 is not expressed in the islets of neither K8$^{+/+}$ nor K8$^{–/–}$ mice. Islets are marked by dotted lines. Scale bar = 50 μm. n >3 mice per genotype.

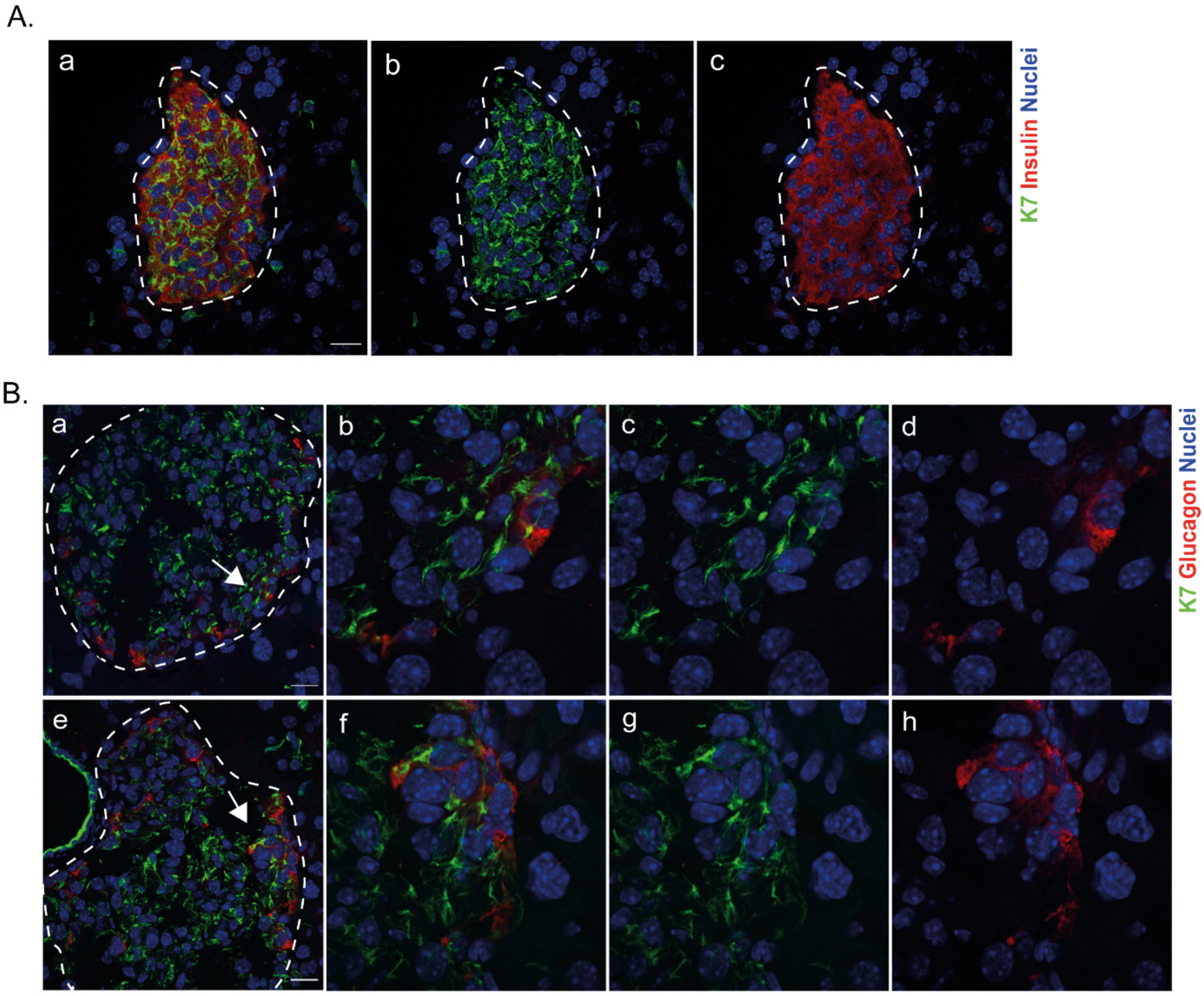

Supplementary Figure S2. Glucagon producing α-cells express K7. A) Pancreatic tissue sections from K8$^{+/+}$ mice were immunostained for K7 (green), insulin (red) and nuclei (blue). The merged image of double staining as well as the individual separate images stained for K7 and insulin (a-c), show the K7 expression in insulin producing β-cells as indicated. B) Immunostained pancreatic tissue sections for K7 (green), glucagon (red) and nuclei (blue). The merged images of double staining (a, e) as well as the higher magnification individual separate images (b-d, f-h), show the K7 expression in glucagon producing α-cells. Islets are marked by dotted lines. Scale bar =20 μm, n = 3 mice.

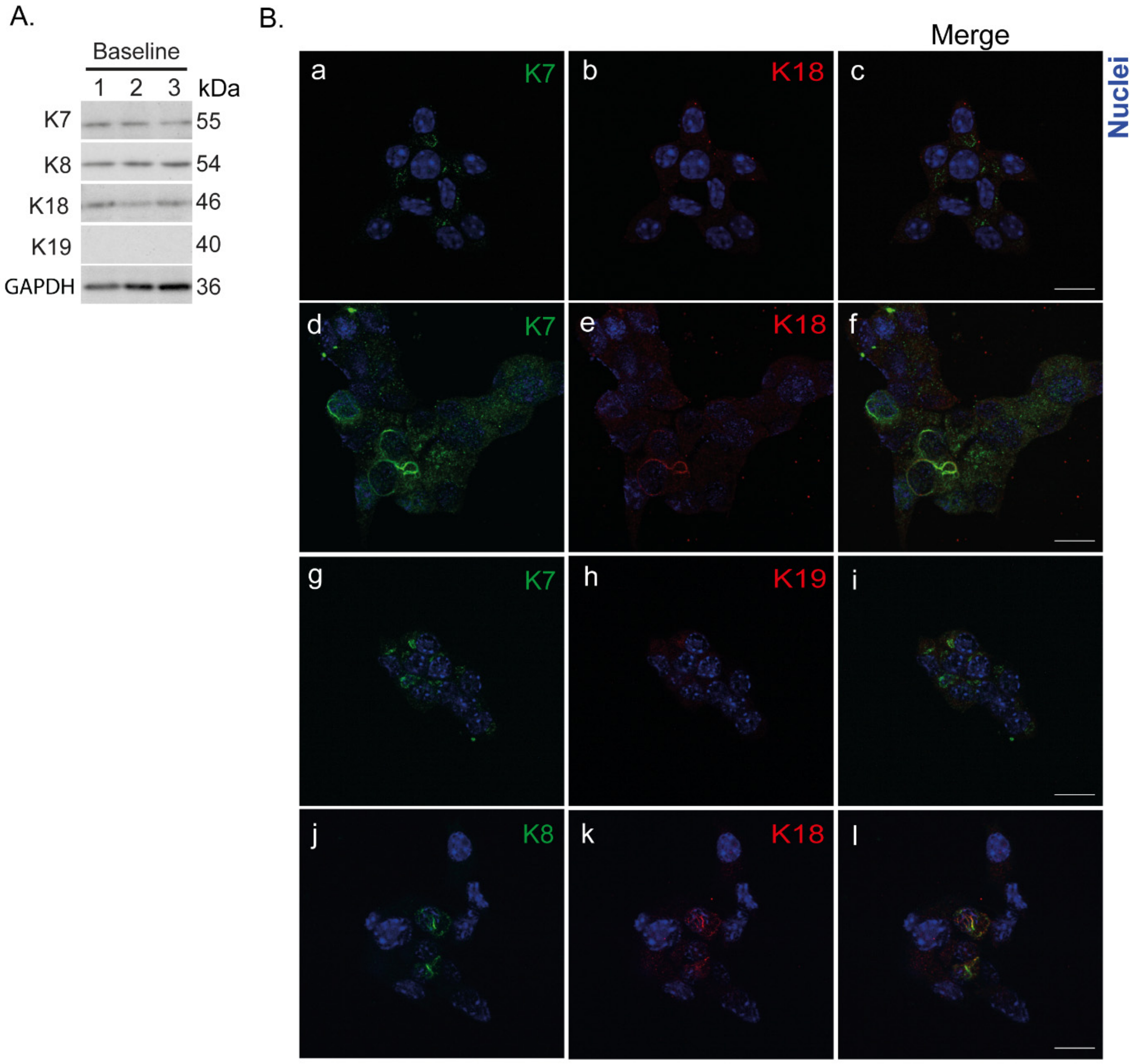

Supplementary Figure S3. MIN6 β-cells express low levels of endogenous K7, K8 and K18 but not K19. A) Protein lysate from MIN6 β-cells (lane 1-3) were immunoblotted for K7, K8, K18, K19 and GAPDH (loading control). B) MIN6 β-cells were immunostained for K7, K8, K18, K19 and nuclei as indicated. The merged images of K7/K18 staining (c, f) as well as the individual separate images (a, b/d, e) show K7 as diffuse non-filamentous fragments and occasionally in filament form. For K7/K19, the individual (g, h) and merged (i) images, show the absence of K19 in MIN6 β-cells while the individual (j, k) and merged (l) images of staining for K8/K18, show these keratins in non-filamentous and filament form. Scale bar = 20 μm.

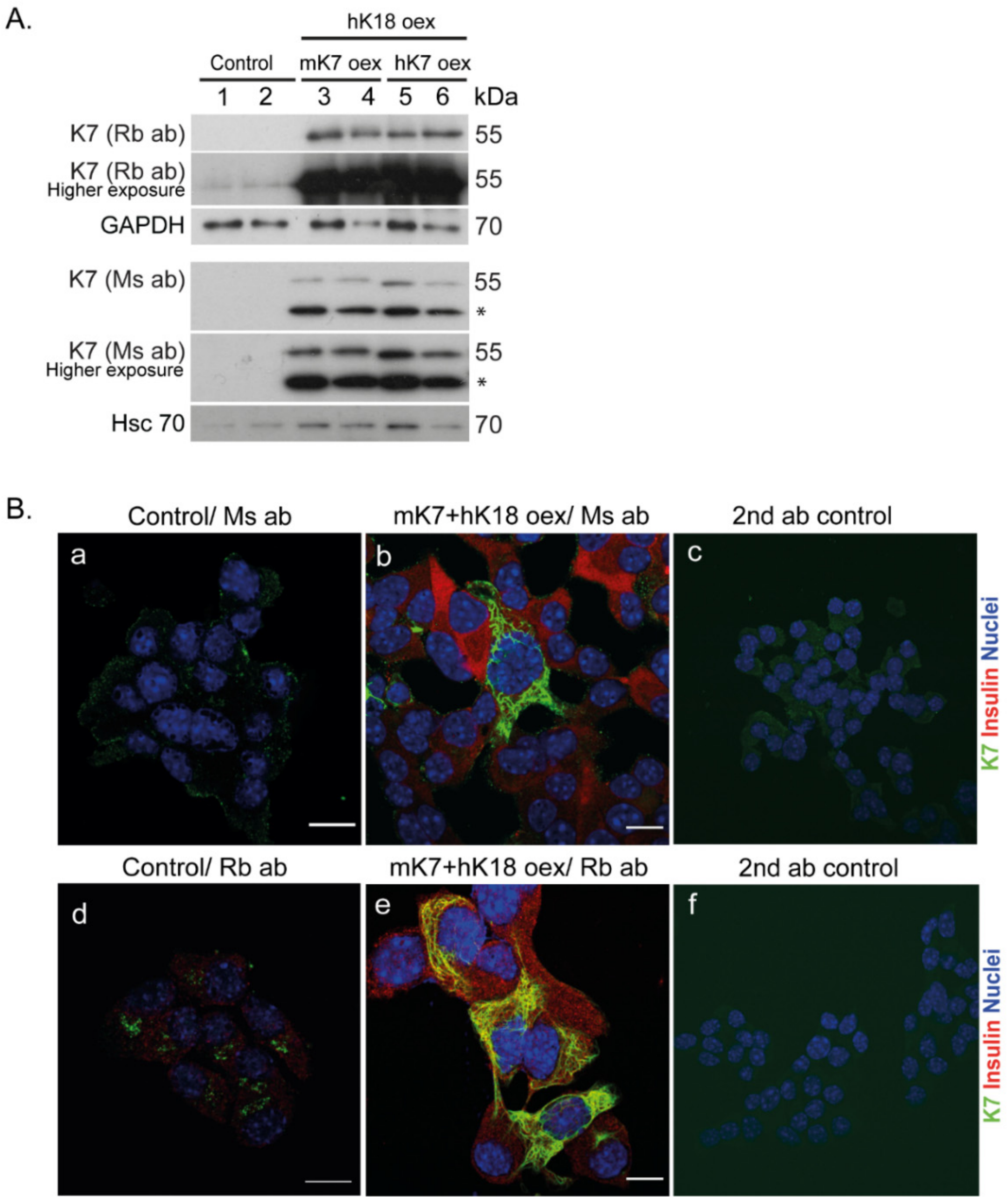

Supplementary Figure S4. Rabbit anti-K7 antibody specifically detects the K7 protein. A) Protein lysate from control MIN6 β-cells (lane 1-2), MIN6 β-cells transfected with mouse K7 (mK7) (lane 3-4) and human K7 (hK7) (lane 5-6), were immunoblotted using both anti-K7 mouse (Ms) and anti-K7 rabbit (Rb) antibodies (ab). hK18 overexpression (oex) was used as the obligate partner for both m/hK7. Both K7 antibodies recognized a 54/55 kDa protein by immunoblotting, for mouse and human K7, respectively. A 55 kDa (K7) band was only observed with the anti-K7 Rb antibody in control MIN6 by over-exposure of the same membrane. * Asterisk indicates an unspecific band. B) Immunostaining of K7 (green), insulin (red) and nuclei (blue) in control MIN6 β-cells using anti-K7 Ms (a) and anti-K7 Rb (d) antibody showed that, only the anti-K7 Rb detects weak endogenous K7 diffuse fragments. Both antibodies detect K7 filaments in MIN6 over-expressing mK7/hK18 (b, e). Secondary antibody (2nd ab) control is shown in (c, f). Scale bar = 20 μm.
